# Novel digital technique for assessing circumferential peri-implant bone height

**DOI:** 10.1186/s40729-024-00583-6

**Published:** 2024-12-19

**Authors:** Armin Sokolowski, Benjamin Gottschalk, Sebastian Glockner, Elisabeth Steyer, Katarina Kalinova, Martin Lorenzoni, Alwin Sokolowski

**Affiliations:** 1 Division of Restorative Dentistry, Periodontology and Prosthodontics, Department of Dental Medicine and Oral Health, Medical University of Graz, Billrothgasse 4, 8010 Graz, Austria; 2 Molecular Biology and Biochemistry, Gottfried Schatz Research Center, Medical University of Graz, Graz, Austria

## Abstract

**Objective:**

To introduce a novel digital technique for precise assessment of peri-implant bone heights, enhancing accuracy and objectivity in dental implantology research.

**Methods:**

This study utilized digital intraoral scans and digitized impressions obtained during implant exposure surgery, combined with computer-aided design (CAD) software, to measure peri-implant bone heights accurately during flap-raising procedures. The peri-implant bone measurements were quantified circumferentially and validated through a comparative analysis of intraoral and extraoral scans.

**Results:**

The technique demonstrated high precision, with a strong correlation (ICC = 0.902) between bone heights determined from intraoral and extraoral scans, highlighting minimal deviations and similar measurement outcomes. This approach enables comprehensive circumferential data and surface area measurements of peri-implant bone levels.

**Conclusion:**

The proposed digital technique provides an objective, reliable method for peri-implant bone height assessment, offering precise, reproducible data that addresses the limitations of traditional probing and conventional imaging methods. This technique has broad applicability in dental implantology research, particularly for assessing peri-implant bone levels when a flap is raised.

## Introduction

The accurate measurement of peri-implant bone is crucial for scientific implantology research focusing on bone remodeling under different surgical protocols, evaluating implant success, and guiding clinical decisions. To evaluate marginal bone loss, it is essential to use radiographic follow-ups and assess mean coronal bone resorption around implants [1–3]. However, challenges arise in comparing mean marginal bone loss data across different research due to varying surgical protocols and measurement techniques [4]. Traditional methods, such as periodontal probes, offer only approximate measurements due to their subjective nature, while radiographic methods provide two-dimensional measurements but face reproducibility limitations [5]. This is particularly relevant when evaluating peri-implant bone tissue at the time of implant placement or during exposure [6]. Lorenzoni et al. [7] highlighted these challenges utilizing periodontal probes with 1-mm calibrations during re-entry surgery to compare bone levels in relation to the implant margin in different loading protocols [7]. Key criteria for implant success, such as the stability of surrounding bone, have been underscored in seminal works like that of Albrektsson et al. [8]. Within implantology, various factors—including physiological or pathological bone remodeling and specific implant designs—significantly influence bone dynamics [9]. However, traditional evaluation methods for implant osseointegration, often relying on submucosal healing and reentry surgery conducted around 3 months post-implantation, may overlook subtle bone changes [10]. As Pawar and Karkar (2020) described, bone remodeling around dental implants is a multifaceted process involving sequences of cell activation, bone resorption, and bone formation, taking approximately 4 months in humans [11]. The novel technique presented in this paper specifically addresses the evaluation of fully exposed implants at the time of placement or exposure, where precise, reproducible, and objective measurements of peri-implant bone heights are essential. As shown by Richert et al. [12], the correct technique for intraoral scanning is crucial [12]. The type of surface being scanned also plays a significant role, as it can influence the accuracy of the scans and the quality of the data for subsequent analysis. Additionally, Vag et al. [13] emphasized that while most modern intraoral scanners are highly capable of producing precise digital impressions, there are still certain situations where the use of lab scanners may be necessary to achieve the desired trueness and precision [13]. Utilizing specialized CAD software, this technique employs digital intraoral scans or digitized conventional impressions to provide accurate data of peri-implant bone heights.

## Technique

### Expose bone

**Fig. 1:**
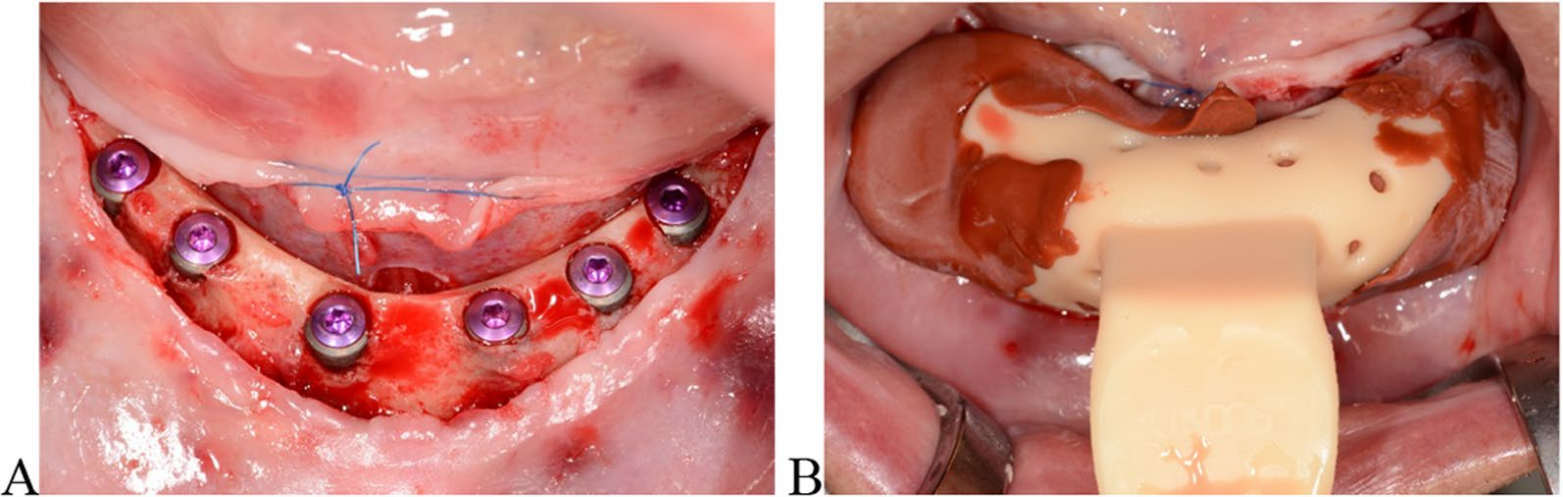
Surgical site with exposed bone. **A** Six implants in the mandible. **B** Intraoperative impression

Begin by exposing the bone in an open flap procedure with buccal and lingual retention sutures. The method can be applied either immediately after implant insertion or during an implant uncovering procedure. Ensure the peri-implant area is clean and minimally bloody (Fig. 1A). Use cover screws inserted into the implants as a reference point for measuring bone height.

### a. Intraoral Scan

**Fig. 2:**
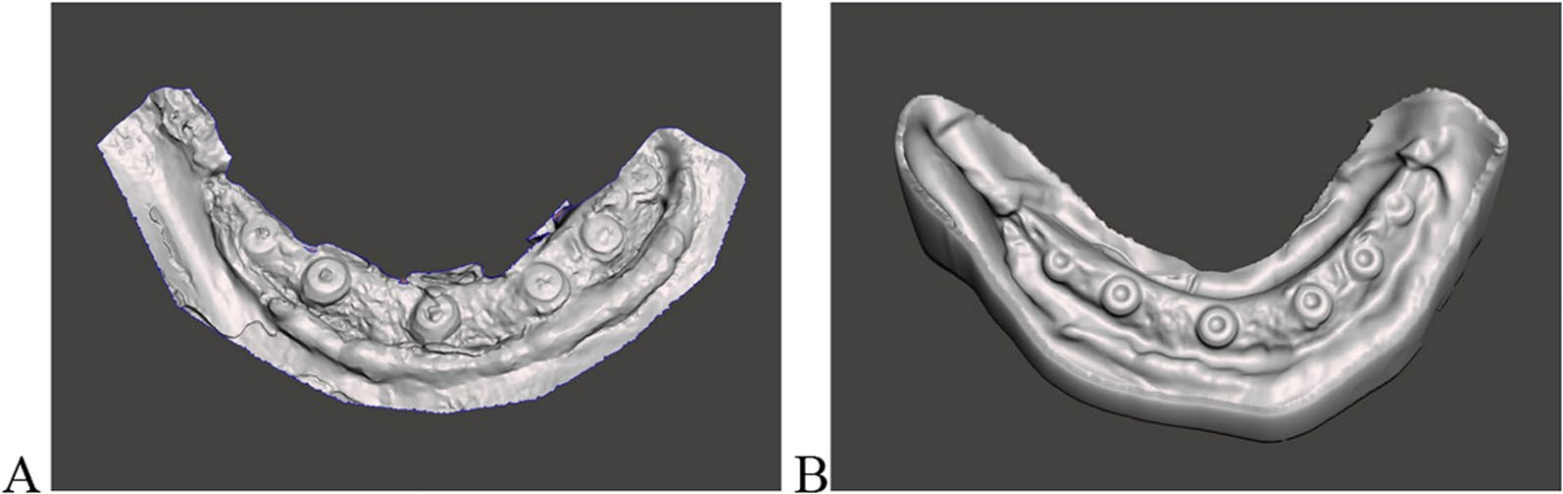
Digital models. **A** Digital model from intraoral scan of surgical site. **B** Digital model from extraoral scan of impression compound

Perform an intraoral scan using an intraoral scanner (Primescan AC, SIRONA Dental Systems GmbH) with an accuracy of 10 ± 2 µm. Capture the bone and implant areas (Fig. 2A).

### b. Intraoperative impressions

Take intraoperative impressions using thermoplastic impression compound (Impression Compound, Kerr). Store the compound in sterile saline, warming it to 55 °C before use. Fill custom trays with the warmed compound, insert them into the surgical site, and cool with sterile saline to set the impression (Fig. 1B). Digitize the impressions using a lab scanner (3Shape D2000, 3Shape) with an accuracy of 5 µm (Fig. 2B).

### Digital analysis

**Fig. 3:**
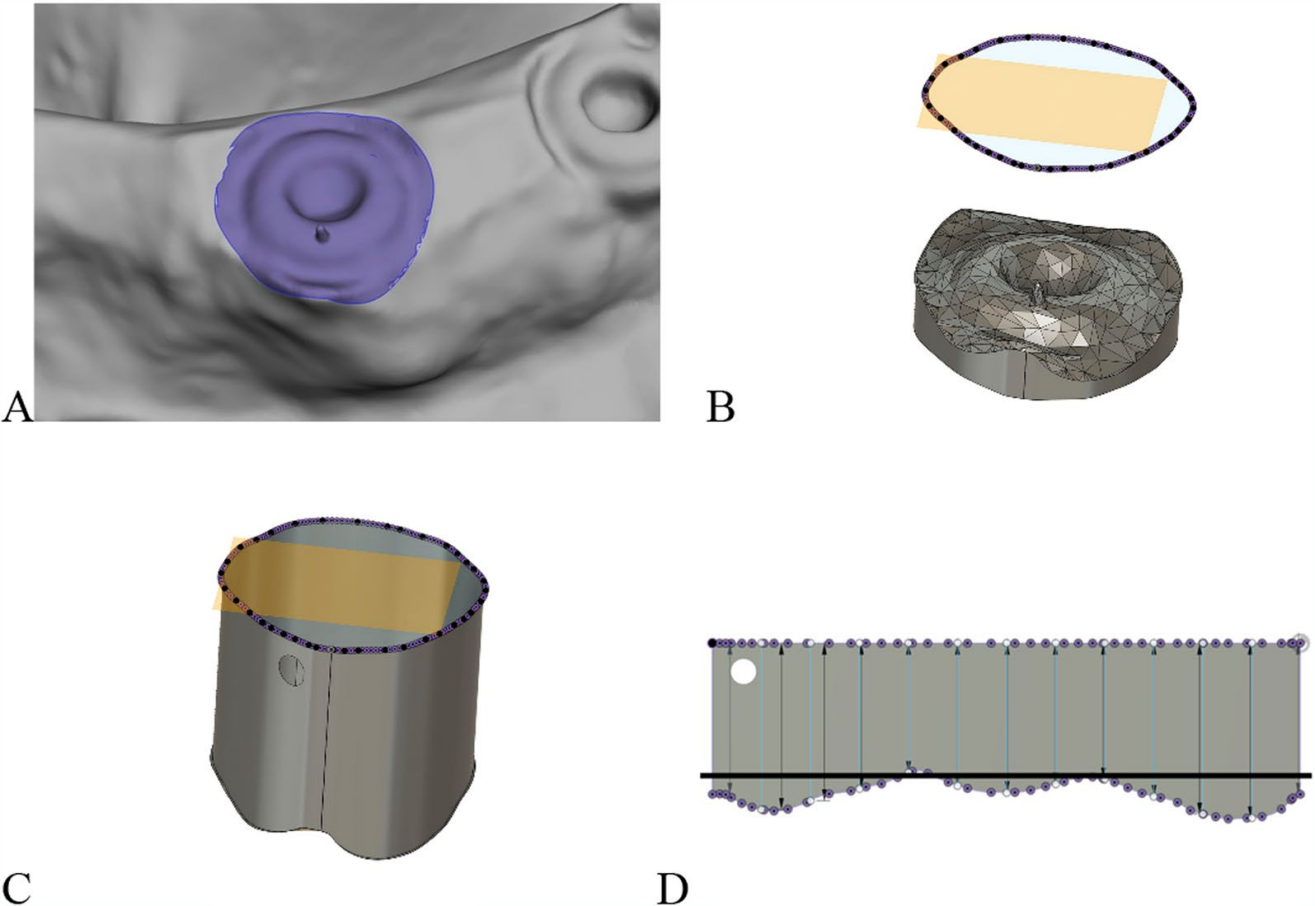
Digital model in CAD software. **A** Implant with cover screw and surrounding area at the first bone-implant contact. **B** Cover screw with surrounding bone and parallel plane (light orange) coronal to cover screw, projection of surrounding bone contour (purple line). **C** Cylinder extending between bone contour and projected plane, buccal side marked by hole. **D** Flattened cylinder, upper boundary: plane shifted coronally from cover screw, lower boundary: bone contour circumferentially around cover screw. Purple dots: node points from STL file triangulation, light blue vertical lines: 12 measurement distances around implant, black horizontal line: level of cover screw

- Load the digital scans into 3 dimensional (3D) mesh modeling software (Meshmixer v3.5, Autodesk) in Standard Triangle Language (STL) format.
- Mark the cover screw and the peri-implant bone at the first bone-implant contact, then export the data as an STL file (Fig. 3A).
- Import the STL files into 3D CAD software (Fusion 360 v2.0.19440, Autodesk).
- Create a parallel plane coronal to the cover screw to capture the entire bone contour (Fig. 3B).
- Construct a cylinder extending from the bone contour to the projected plane (Fig. 3C).
- Flatten the cylinder using the "Create Flat Pattern" function (Fig. 3D).
- Adjust the plane to the height of the cover screw to interpret regions as either bone recession (negative values) or bone excess (positive values) (Fig. 3D).

### Interpretation

The values should be read vertically to make circumferential assessments around the implant’s cover screw. Furthermore, the surface area of bone loss can be calculated by comparing scans from two different time points.

## Discussion

**Fig. 4:**
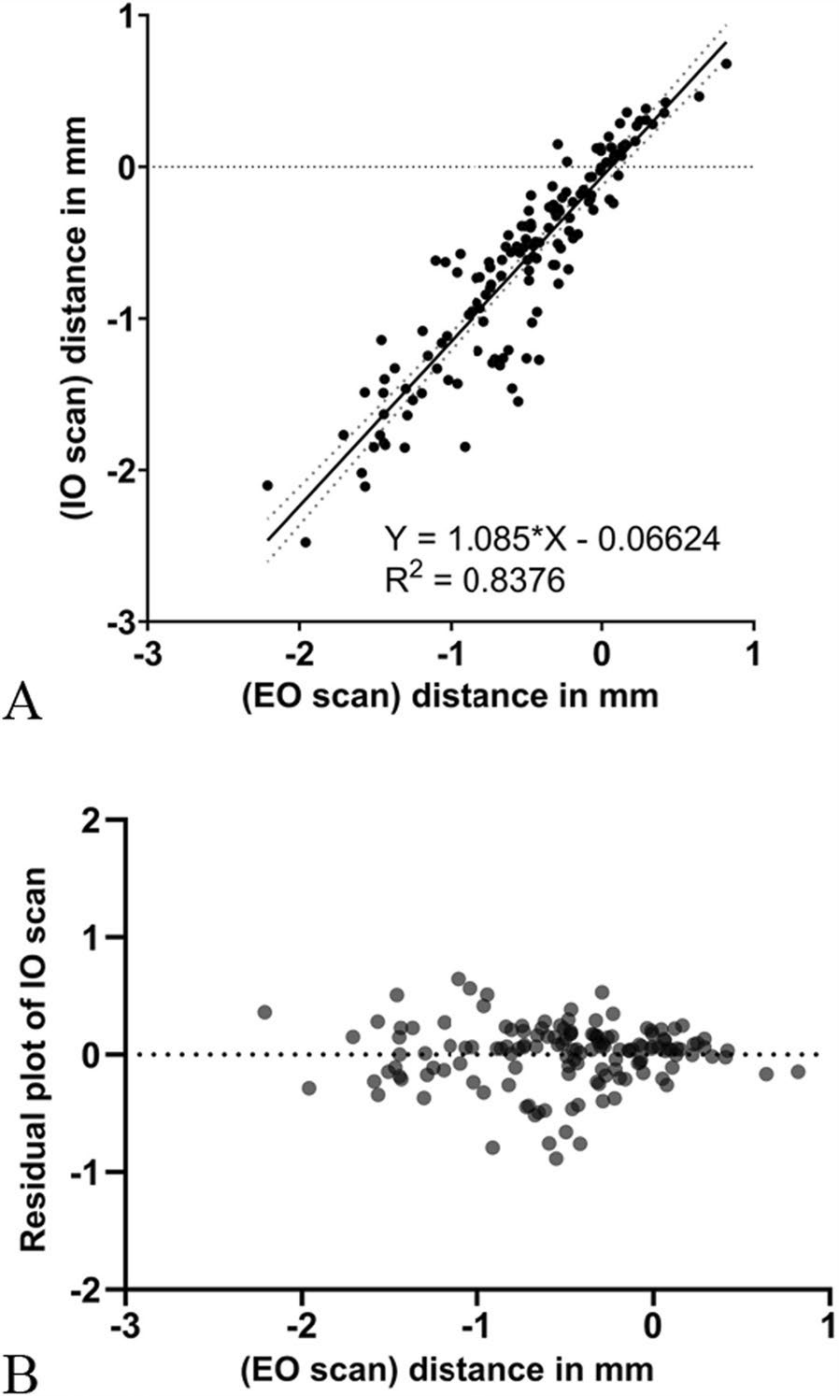
**A** Linear regression curve comparing measurement values from IO scan and EO scan (R^2^ = 0.8376). **B** Residual plot illustrating extent of deviation in IO scan values from EO scan values, both positive and negative

**Fig. 5:**
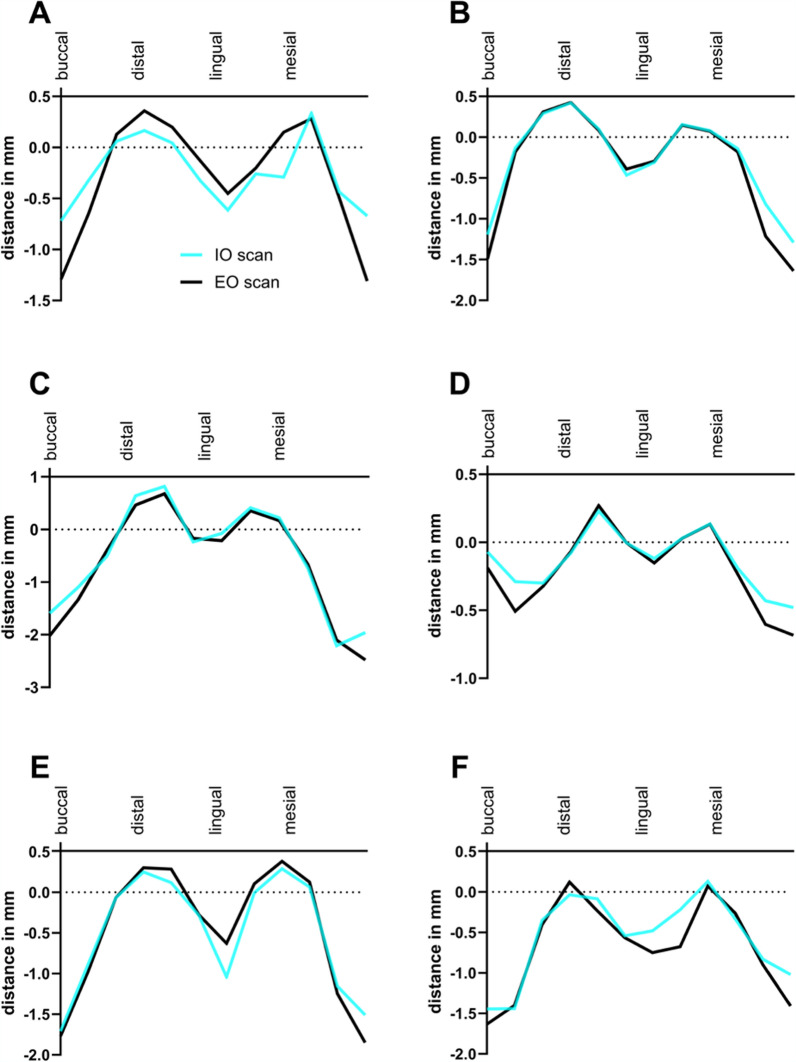
Curves representing peri-implant bone contours of six implants (**A**–**F**). IO scan – intraoral scan (light blue), *EO* scan – extraoral scan (black)

This novel technique, which incorporates both intraoral and extraoral scanning, provides a method for precise and reproducible measurements of peri-implant bone heights whenever a flap is raised during implant surgery. The technique allows for assessing bone height at different time points, such as baseline and re-entry, providing valuable insights into bone remodeling processes. The reliability of this technique was confirmed by a comparative analysis between intraoral (IO) and extraoral (EO) scans in a patient who received six implants (OsseoSpeed EV 3.6 -11 mm, Astra Tech Implant System, Dentsply Sirona) in the mandible. The high correlation observed between the two scanning methods (ICC = 0.902) and a slope of the linear regression curve close to 1 (R^2^ = 0.8376, Fig. 4A) suggests that both IO and EO scans yield consistent measurement results. Furthermore, the residual plots showed a narrow spread with deviations equally distributed between positive and negative values (Fig. 4B), indicating high similarity between the methods. The peri-implant bone contours of all six implants captured by both techniques also demonstrated similar patterns (Fig. 5). This technique allows for detailed visualization of subtle changes in the peri-implant bone profile.

The selection of the surrounding bone area was designed to closely replicate the clinical scenario of using a periodontal probe for assessing peri-implant bone levels. By marking the bone area at the first bone-implant contact, we aimed to standardize the measurement process and ensure consistency. The findings demonstrate the technique’s potential to enhance the accuracy and reliability of peri-implant bone measurements, providing a comprehensive view of the peri-implant bone. This is particularly essential for studies that require detailed data on bone remodeling and marginal bone loss. The ability to measure circumferential bone continuously around the implant facilitates a detailed analysis of bone loss or gain, and enables the assessment of bone area changes over time by comparing measurements from different time points. However, intraoral scans within the surgical site can pose challenges, particularly due to moving and bleeding tissues, which may necessitate a longer scanning procedure to capture all peri-implant bone areas clearly (Fig. 2A). The difficulty is further amplified in edentulous mandibles with multiple implants, where the absence of fixed reference points complicates optical data collection using intraoral scanners. In contrast, intraoperative impressions using Kerr impression compound are quicker and more straightforward, allowing for an uncomplicated post-operative scan with the lab scanner. This method results in cleaner and more uniform STL files with fewer artifacts, providing more detailed images (Fig. 2B). Conventional impressions can displace blood and saliva, leading to a more homogeneous result. Despite the challenges associated with intraoral scanning, this technique offers significant advantages. However, it does require the use of specialized software for data analysis, which necessitates a certain level of technical expertise.

## Conclusions

This investigation presents a method using scanned surfaces of placed implants and surrounding bone, combined with CAD software, to precisely measure peri-implant bone heights. The technique allows for assessment at various time points, making it suitable for long-term studies and evaluating different healing protocols. The indirect method, involving intraoperative impressions and EO scanning, proved easier to apply clinically and produced higher-resolution scans with better bone visibility. While this technique provides precise data and detects subtle changes in the peri-implant bone profile, it requires sufficient exposure of implants and adjacent bone through an open flap. Despite this limitation, it offers broad applicability in oral implantology research, particularly for studying bone remodeling and marginal bone loss over time.

## Data Availability

No datasets were generated or analysed during the current study.

## Abbreviations

CAD: Computer-Aided design
ICC: Intraclass correlation coefficient
3D: Three-dimensional
STL: Standard tessellation language
IO-Scans: Intraoral scans
EO-Scans: Extraoral scans
